# CRISPR/Cas12a Technology Combined With RPA for Rapid and Portable SFTSV Detection

**DOI:** 10.3389/fmicb.2022.754995

**Published:** 2022-01-25

**Authors:** Mengqian Huang, Sihua Liu, Yanan Xu, Aqian Li, Wei Wu, Mifang Liang, Guoyu Niu, Zhiyun Wang, Tao Wang

**Affiliations:** ^1^School of Life Sciences, Tianjin University, Tianjin, China; ^2^National Institute for Viral Disease Control and Prevention, Chinese Center for Disease Control and Prevention, Beijing, China; ^3^School of Public Health, Weifang Medical University, Weifang, China; ^4^School of Environmental Science and Engineering, Tianjin University, Tianjin, China; ^5^Institute of Tianjin Key Laboratory of Function and Application of Biological Macromolecular Structures, Tianjin, China

**Keywords:** SFTSV, CRISPR/Cas12a, RPA, immunochromatographic strips, detection

## Abstract

Severe fever with thrombocytopenia syndrome virus (SFTSV) is a new tick-borne pathogen that can cause severe hemorrhagic fever. Fever with thrombocytopenia syndrome caused by SFTSV is a new infectious disease that has posed a great threat to public health. Therefore, a fast, sensitive, low-cost, and field-deployable detection method for diagnosing SFTSV is essential for virus surveillance and control. In this study, we developed a rapid, highly sensitive, instrument-flexible SFTSV detection method that utilizes recombinase polymerase amplification and the CRISPR/Cas12a system. We found that three copies of the L gene from the SFTSV genome per reaction were enough to ensure stable detection within 40 min. The assay clearly showed no cross-reactivity with other RNA viruses. Additionally, our method demonstrated 100% agreement with Q-PCR detection results for SFTSV in 46 clinical samples. We simplified the requirements for on-site detection instruments by combining the CRISPR/Cas12a tool and immunochromatographic strips to create a system that can reliably detect one copy/μl sample of the L gene, which showed extremely high sensitivity and specificity for detecting the virus. Taken together, these findings indicate that the new SFTSV detection method is a powerful and effective tool for on-site detection, which can contribute to diagnosing SFTSV quickly and sensitively.

## Introduction

The spread of a new bunyavirus, severe fever with thrombocytopenia syndrome virus (SFTSV), has resulted in more than 11,986 cases diagnosed clinically and a total mortality rate of 5.1% in 18 provinces of China (Zhan et al., 2017). In addition, cases of SFTS have also been reported in Vietnam, Japan, and South Korea, and the average mortality rate in Japan and South Korea is as high as 6–30% (Li et al., 2021; Xu et al., 2021; Zhou and Yu, 2021). Individuals of any age are susceptible to SFTSV; most of them live in mountainous and hilly areas and are predominantly engaged in agricultural work, such as planting crops or tea (Zhan et al., 2017; Chen et al., 2019). The virus has attracted great concern about public health safety worldwide. Generally, patients infected with SFTSV experience fever, leukopenia and thrombocytopenia, bleeding, gastrointestinal symptoms, and multiple organ failure (Bopp et al., 2020). Since SFTSV poses a great threat to public health and there is no vaccine or drug for clinical prevention or treatment of humans infected by SFTSV (Reece et al., 2018), it is essential to diagnose suspected cases accurately in the early stage of infection to avoid widespread infection (Li et al., 2021).

SFTSV is an enveloped, single-stranded, negative-sense RNA virus belonging to the family phlebovirus. Its genome consists of three single-stranded negative-strand RNA fragments: large (L), medium (M), and small (S). The L segment contains 6,368 nucleotides and encodes RNA-dependent RNA polymerase (RdRp), which mediates viral RNA replication and mRNA synthesis (Saijo, 2018b; Bopp et al., 2020; Li et al., 2021). The M segment consists of 3,378 nucleotides, contains an open reading frame, and encodes viral envelope glycoproteins (glycoprotein N Gn and glycoprotein C Gc), which play an important role in virus assembly and virus particle formation. The S fragment consists of 1,744 nucleotides and functions as ambisense RNA (Saijo, 2018a; Bopp et al., 2020); it contains two reverse reading frames, which encode the virus nucleoprotein (NP) and virus non-structural protein (NSs) (Zhan et al., 2017). The S segment is the most conserved gene; the L segment also has a conserved region and can be used as a target for testing (Vogel et al., 2020). Accordingly, the L, M, and S genes of SFTSV could all be used for nucleic acid detection; the detection of these three genes reveals that there are no obvious differences in sensitivity or specificity by the L, M, and S segments (Lee et al., 2020).

A series of molecular detection assays targeting the SFTSV genome have been developed, based on methods, such as PCR, real-time PCR (RT-PCR; Sun et al., 2012), and loop-mediated isothermal amplification (LAMP; Yang et al., 2012). However, most of these methods show low sensitivity or a high false-negative rate. Recently, the CRISPR/Cas endonuclease detection system has shown a broad prospect in the field of molecular diagnostics due to its high sensitivity and specificity and low cost (Zetsche et al., 2015). Cas9, which recognizes seven or eight of 20 bases in gRNA, is the first and most commonly used enzyme in CRISPR technology. However, because it can cut a DNA sequence similar to the target DNA – which is known as the missing target effect – Cas9 is not an ideal candidate tool to detect SFTSV. Compared with Cas9, Cas12a can recognize up to 18 bases before fully combining with the target DNA, which results in a lower rate of mistakes and higher security. Furthermore, Cas12a is considered a good choice to solve the known shortcomings of CRISPR. Previous studies have shown that Cas12a is activated to arbitrarily cut single-strand DNA (ssDNA) after recognizing double-strand DNA (dsDNA) (Li et al., 2018; Qian et al., 2019). Additionally, the CRISPR/Cas12a technology has been successfully applied to detect bacteria (Pang et al., 2019; Li et al., 2020), viruses (Yuan et al., 2020), plants (Wu et al., 2020), and exosomes (Zhao et al., 2020). This technology is most widely used for virus detection, including the African swine fever virus (ASFV) (He et al., 2020), human papillomavirus 16 (HPV16) (Dai et al., 2019), parvovirus B19 (PB-19), white spot syndrome virus (Chaijarasphong et al., 2019), and other viruses.

In this study, we developed a nucleic acid molecular detection system targeting the L gene for SFTSV detection with CRISPR/Cas12a technology. This method has the advantages of high sensitivity and specificity of CRISPR/Cas12a technology and can be used for on-site testing with a portable testing instrument or immunochromatographic strips.

## Materials and Methods

### Human Clinical Sample Collection and RNA Preparation

Clinical RNA samples used in this study were obtained from the Chinese Center for Disease Control and Prevention (Beijing) and Weifang Medical University (Weifang). Clinical samples used in this study were collected and treated in strict accordance with the standard operating procedures for SFTSV recommended by the WHO. 30 sample treatments were conducted in the Laboratory of China, Beijing, and 16 sample treatments were conducted in the Laboratory of China, Weifang. In accordance with the requirements of the National Health Commission of China, SFTSV was inactivated in a BSL-2 laboratory at 60°C for 30 min. Additionally, the viral RNA was extracted in a BSL-2 laboratory using a QIAamp Viral RNA Kit in accordance with the manufacturer’s instructions (QIAGEN, cat. no. 52906).

### Nucleic Acid Preparation

The SFTSV HB29 segment L was cloned into the VR1012 vector. The RNA of the L gene was transcribed with VR-SFSTV-L as a template using the T7 Transcription Kit (VIEWSOLID BIOTECH, cat. no. VK010) in accordance with the manufacturer’s instructions. The concentration was determined using Nanodrop, and the samples were stored at −80°C until use. The crRNA, primer, ssDNA FQ probe, and ssDNA FB probe were also synthesized by GenScript. The sequences are listed in Table 1.

**Table 1:** List of primers and crRNA.

	Sequence (5′-3′)
ssDNA-FQ reporters	IAB-TTATT-BHQ-1
ssDNA-FB reporters	FAM-TTATT-Biotin
crRNA-1	**UAAUUUCUACUAAGUGUAGAU** AGAAUUGGGGAAUGUUCCCUCCAC
Primer 1-F1	GCTTGAGGCTATTAGTAGGGCA
Primer 1-R2	TGTAAGTTCGCCCTTTGTCCAT
Primer 2-F2	CAGTCTGTTTGAGCCTAACATT
Primer 2-R2	TGAATAGTTTTCTCAGGCTTTA
Primer 3-F3	CTATCATTCTTCAGTCTGTTTG
Primer 3-R3	GGTAGTGACTGTCGCAAAGATC
Primer 4-F1	GCTTGAGGCTATTAGTAGGGCA
Primer 4-R4	GCTTTGAGGGTCTTGGTGATGA
Primer 5-F2	CAGTCTGTTTGAGCCTAACATT
Primer 5-R5	AGCATAGGCCTCTACCTCTTTT
Primer 6-F3	CTATCATTCTTCAGTCTGTTTG
Primer 6-R6	AGTCAACATCAGCTGGTGTTGAG
crRNA-2	**UAAUUUCUACUAAGUGUAGAU** GAAAAGUUGCUUGUAGCUUUCAUG
Primer-F7	GTGTCCTGAAAGAGAITGGGAC
Primer-R7	GTACCACATAACCCCCTTCTCA

The bold values indicate the binding sequence of crRNA to Cas12a, which is a universal sequence.

For the extraction of viral RNA, 140 μl of 10^6^ PFU/ml SFTSV strain HB29, 10^4^ PFU/ml influenza virus strain PR8, and 10^6^ PFU/ml human enterovirus D68 (EV-D68) and human enterovirus 71 (EV71) were used. Next, reverse transcription of the viral RNA was performed using a commercial reverse transcription First-Strand cDNA Synthesis SuperMix (TRANS, cat. no. AH321-01) in accordance with the manufacturer’s instructions. Finally, 4 μl of cDNA was added to each detection system for the specificity test.

### Human Enterovirus 71 Culture

Enterovirus 71 (EV71) was propagated in rhabdomyosarcoma (RD) cells. After 72 h, the virus was harvested, and then, the cells were repeatedly frozen and thawed to release the virus. The supernatant and the frozen and thawed cells were mixed evenly, centrifuged, and stored at −80°C.

### Human Enterovirus D68 Culture

Enterovirus D68 (EV-D68) was propagated in RD cells. After 72 h, the virus was harvested, and then, the cells were repeatedly frozen and thawed to release the virus. The supernatant and the frozen and thawed cells were mixed evenly, centrifuged, and stored at −80°C.

### Influenza Virus PR8 Culture

Influenza virus PR8 was propagated in Madin–Darby Canine Kidney (MDCK) cells. The supernatant was harvested 3 days post-infection, centrifuged, and stored at −80°C.

### Quantitative Real-Time PCR

The quantitative real-time PCR (Q-PCR) detection of SFTSV was carried out using a Fast Advanced Master Mix approved for the market in accordance with the manufacturer’s instructions (Applied Biosystems, cat. no. 4444556). In brief, we prepared single-tube PCRs containing 10 μl TaqMan® Fast Advanced MasterMix (2×), 0.4 μl 10 μM SFTSV-L forward primer, 0.4 μl 10 μM SFTSV-L reverse primer, 0.4 μl 10 μM SFTSV-L probe (Sun et al., 2012), 6.8 μl DEPC water, and 2 μl sample DNA. The amplification conditions used included an initial denaturation step of 45°C for 2 min and 95°C for 10 min, followed by 40 cycles of 95°C for 15 s and 60°C for 60 s. If the cycle threshold (Ct) value was ≤40, the sample was judged as SFTSV-positive.

### CRISPR/Cas12a Detection Reaction

Isothermal amplification of the L gene was performed using a commercial recombinase polymerase amplification (RPA) kit (TwistAmp, cat. no. TABAS03KIT) in accordance with the manufacturer’s instructions. In brief, a 50-μl reaction assembled with a 4 μl DNA sample (4 μl DEPC water as a blank group), 2.5 μl forward primer and reverse primer (10 μM), 29.5 μl Primer Free Rehydration buffer, 9 μl DEPC water, and 2 μl activator was incubated at 39°C for 20 min. Next, 4 μl crRNA (1 μM) and 3 μl buffer were incubated with 1 μl Cas12a (1 μM; New England Biolabs cat. no. M0653S) at 25°C for 20 min and fully combined. Then, a 20 μl RPA reaction system and 2 μl ssDNA FQ probe (10 μM, labeled with FAM and BHQ1) were transferred to the CRISPR/Cas12a cleavage assay. Reactions were incubated in a GS8 Isothermal Cycler (GenDX, cat. no. GS8) for 20 min at 37°C, with fluorescence measurements taken every 30 s.

For immunochromatographic strip detection reactions, the 20-μl RPA reaction system and 2 μl ssDNA FB probe (100 μM, labeled with FAM and biotin) were transferred to the CRISPR/Cas12a cleavage assay. The reaction was performed at 37°C for 20 min, after which the CRISPR/Cas12a detection reaction was diluted 1:2 in detection buffer, and then, the strips (Milenia Biotec, cat. no. MGDH1) were inserted and incubated at room temperature for 1 min. The strips were then removed and photographed with a camera. To quantify and visualize the strips, the band density was analyzed by ImageJ.

### Statistics and Reproducibility

All results were analyzed with GraphPad Prism software and are presented as mean ± SD. The unpaired *t* test was applied for the statistical analysis in GraphPad Software Prism 8, *p < 0.05; **p < 0.01; and ***p < 0.001.

## Results

### Schematic Diagram of SFTSV Detection Based on CRISPR/Cas12a

**Figure 1:**
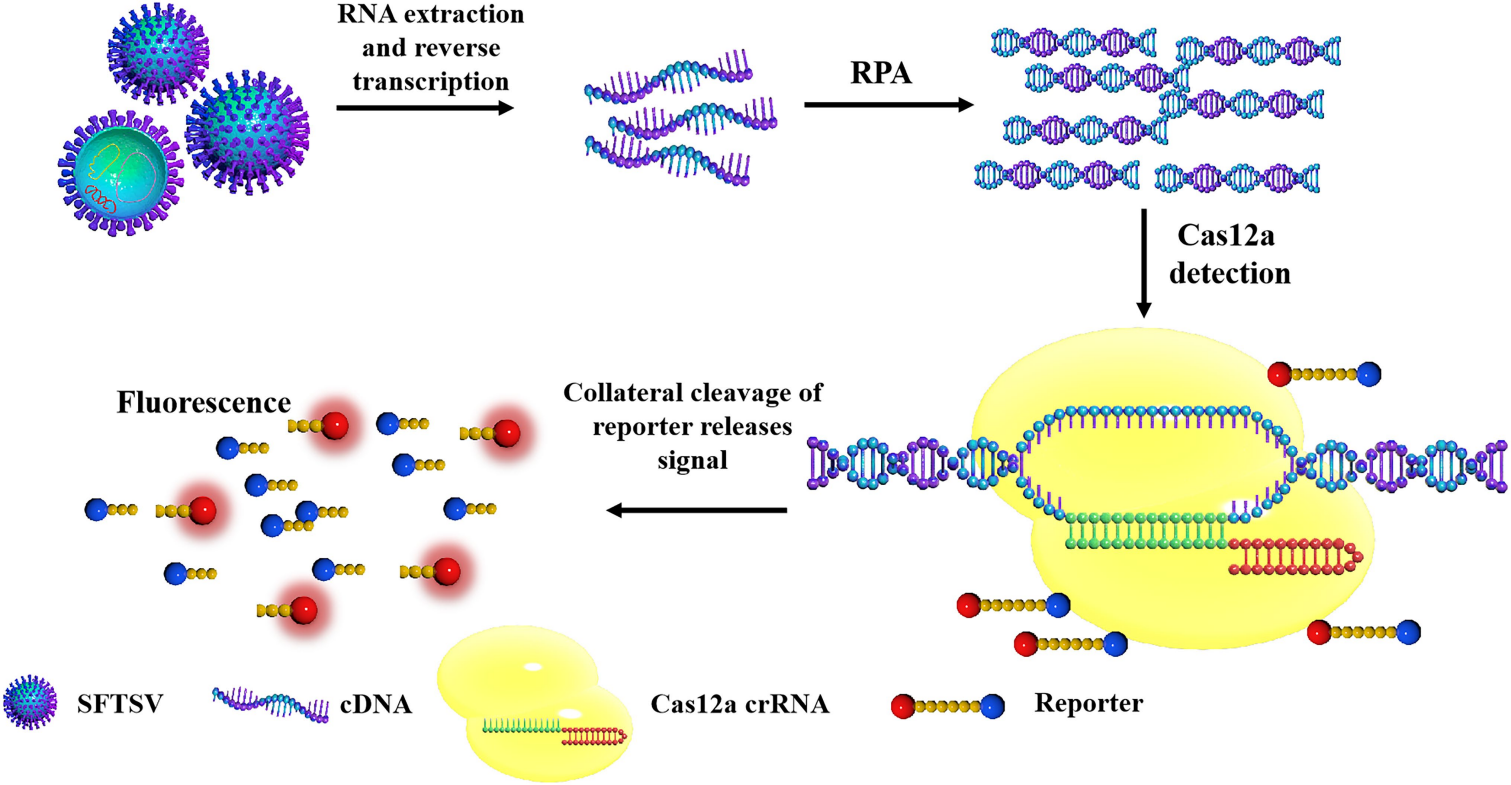
The schematic diagram of severe fever with thrombocytopenia syndrome virus (SFTSV) detection based on CRISPR/Cas12a. The SFTSV-DNA standard substance or SFTSV-DNA extracted from the virus is amplified by recombinase polymerase amplification (RPA). After Cas12a is combined with the PAM sequence, the Cas12a endonuclease is activated and the activated Cas12a cuts the target DNA, the non-specific ssDNA in the system is trans-cut to separate the reporter and the quenching group on the ssDNA, and the reporter emits strong fluorescence.

The principles of the CRISPR/Cas12a-based SFTSV detection system are shown in Figure 1. First, the target gene fragment is amplified by an RPA kit. Under a constant temperature of 39°C, the DNA recombinase capable of binding oligonucleotide primers and specific nucleic acid amplification primers forms protein–DNA complexes in RPA. The complex can search for homologous sequences in the SFTSV genome in the system and amplifies the DNA. The RPA system can amplify target genes exponentially within 20 min. Then, the Cas12a–crRNA complex is added to the amplification system. According to the principle of base complementary pairing, crRNA guides Cas12a to bind to the SFTSV genome. After Cas12a is combined with the PAM sequence, the Cas12a endonuclease is activated; the activated Cas12a cuts the target DNA; the non-specific ssDNA-FQ reporters in the system and the quenching group on the ssDNA are trans-cut, and the reporter emits strong fluorescence. GS8 Isothermal Cycle is a portable fluorescence constant-temperature amplification instrument. It is used to collect fluorescent signals, thereby providing the detection results. The machine can automatically identify positive samples and display the positive signal detection time by calculating the difference in fluorescence values. Compared with the Q-PCR instrument and multilabel plate readers, the GS8 Isothermal Cycle is small and easy to carry and has gradient heating and a built-in battery, which offers great advantages for on-site detection.

### Establishment of SFTSV Nucleic Acid Detection Method Based on CRISPR/Cas12a

**Figure 2:**
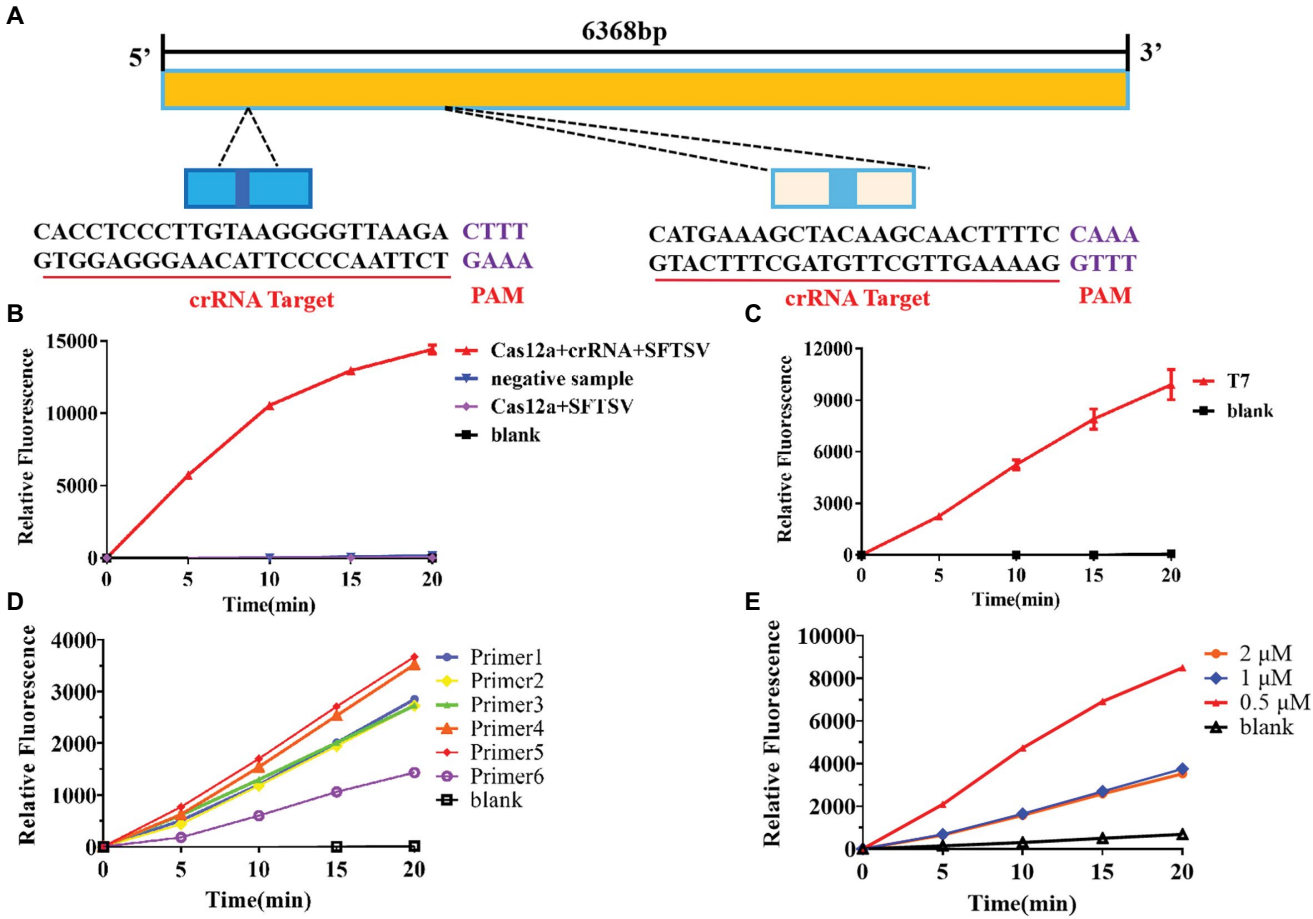
Establishment of the SFTSV nucleic acid detection method based on CRISPR/Cas12a. **(A)** According to the gene sequence of SFTSV-HB29 on NCBI, two crRNAs were designed on the L segment of the conserved region. **(B)** The Cas12a + crRNA + SFTSV plasmid, Cas12a + crRNA + negative sample, Cas12a + SFTSV, and blank were subjected to RPA, and the CRISPR/Cas12a system was used for detection. **(C)** After *in vitro* SFTSV plasmid T7 transcription, RPA and the CRISPR/Cas12a system were used for detection. **(D)** There were 1,000 copies/μl DNA (L gene, final concentration) amplified by RPA with six pairs of primers, and the amplification efficiency of the six pairs of primers was detected by CRISPR/Cas12a assay. **(E)** The 100 copies/μl DNA (L gene, final concentration) was detected by the CRISPR/Cas12a system at 0.5, 1, and 2 μM KCl for 20 min.

Based on the sequence provided by the NCBI, we designed a specific crRNA for L (Figure 2A). CrRNA-1 can be combined with the crRNA-1 target for SFTSV detection, and crRNA-2 can be combined with the crRNA-2 target for SFTSV detection. We used crRNA-1 to establish a CRISPR/Cas12a-based SFTSV nucleic acid detection method. First, we verified the entire CRISPR/Cas12a system (Figure 2B). We found that Cas12a nuclease was activated, and the reporter was only activated when Cas12a, crRNA, and SFTSV were present in the system. Subsequently, we transcribed the plasmid containing the SFTSV L segment by T7 RNA polymerase *in vitro* and obtained the SFTSV-RNA standard. The CRISPR/Cas12a detection of SFTSV showed a significant increase in the fluorescence signal (Figure 2C). At the same time, six pairs of primers were designed to improve the amplification efficiency of RPA. The final concentration of 1,000 copies/μl sample was increased by RPA at 39°C for 20 min, and then, samples were added to the CRISPR/Cas12a system. The fluorescence detection results showed that the primer 5 group resulted in a stronger fluorescence signal (Figure 2D) and a shorter positive judgment time (Table 2). Subsequently, we explored the effect of ion concentration on amplification efficiency. The final concentration of 1,000 copies/μl samples was detected by CRISPR/Cas12a at 0.5, 1, and 2 μM KCl for 20 min. Through the intensity analysis of the fluorescence signal, 2 μM KCl was selected as the optimal ion concentration (Figure 2E). All subsequent experiments were amplified with primer 5 at 39°C for 20 min and detected with 2 μM KCl unless otherwise specified.

**Table 2:** Positive judgment time of primer selection.

Sample name	Check out time	Result
Primer1	16:00	Positive
Primer2	16:30	Positive
Primer3	16:30	Positive
Primer4	13:30	Positive
Primer5	13:00	Positive
Primer6	15:30	Positive
Blank		Negative

### Specificity and Sensitivity Evaluation of the CRISPR/Cas12a Detection System

**Figure 3:**
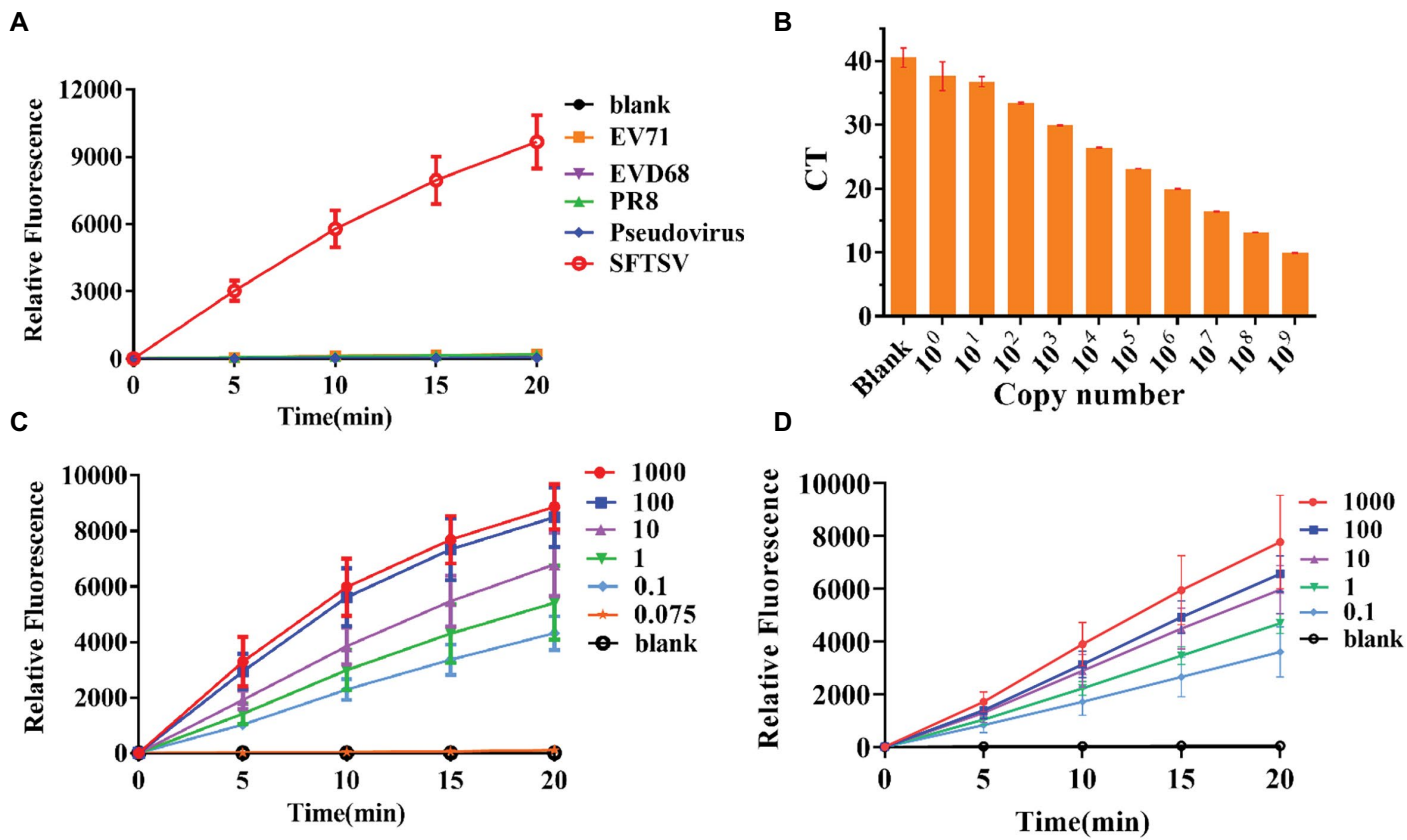
Specificity and sensitivity evaluation of the CRISPR/Cas12a detection system. **(A)** The DNA of SFTSV (1,000 copies/μl, L gene), influenza virus strain PR8, EV-D68, EV71, and pseudovirus was amplified by RPA and detected by the CRISPR/Cas12a system. **(B)** Quantitative real-time PCR (Q-PCR) detection sensitivity evaluation. **(C,D)** The sensitivity of CRISPR/Cas12a combined with RPA for detection of standard substance and virus.

To further test the specificity of the system, we extracted RNA from the influenza virus strain PR8, EV-D68, and EV71. Compared with these viral RNAs, the final RNA concentration of 1,000 copies/μl of the SFTSV L gene was increased by amplification at the same time and the gene was detected using the CRISPR/Cas12a system and crRNA-1. The results showed that positive signals could be detected in the SFTSV samples within 20 min, even if the amount of template RNA in the control group was much higher than the amount of SFTSV (Figure 3A). Our detection system only targeted the SFTSV L genome nucleic acid; it could not detect genomic nucleic acid sequences of other viruses. Thus, the SFTSV CRISPR/Cas12a detection system showed great specificity.

Thus far, Q-PCR is considered the “gold standard” for SFTSV diagnosis due to its high sensitivity and specificity. However, Q-PCR relies on expensive instruments and well-trained personnel, and it might generate a small percentage of false-negative results. We used a fluorescence Q-PCR method to detect SFTSV nucleic acid (Sun et al., 2012) with a detection limit of 1 copy/μl and found that less than 1 copy/μl samples could not be stably tested as positive results (Figure 3B). However, the CRISPR/Cas12a detection system was able to stably detect even 0.1 copies/μl sample of L gene standard substance (Figure 3C) and 0.1 copies/μl sample of the SFTSV L gene (Figure 3D) within 20 min, with a fluorescence signal five times higher than that of the blank group. The results of the significance analysis are shown in Supplementary Figure 1. Subsequently, we infected Vero cells with SFTSV at a multiplicity of infection (MOI) of 0.01, 0.1, and 1. After 24 h, the cells were harvested and RNA was extracted and tested (Supplementary Figure 2). At all three concentrations, the virus was well detected after infection.

### SFTSV Typing Detection Based on CRISPR/Cas12a

**Figure 4:**
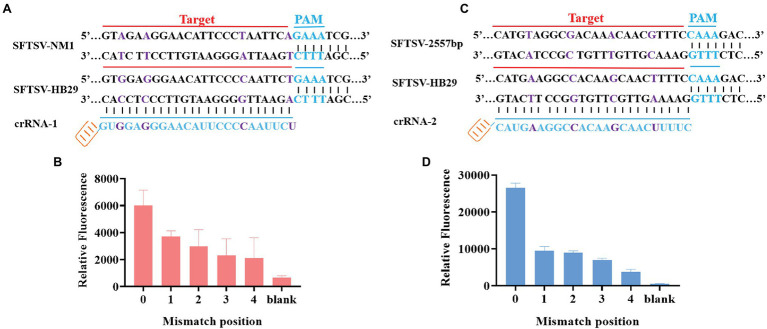
SFTSV typing detection based on CRISPR/Cas12a. **(A)** The SFTSV-HB29 strain and the SFTSV-NM1 strain differ by four bases in the crRNA-1 position. **(B)** The SFTSV-HB29 strain plasmid was detected after one, two, three, and four point mutations in the crRNA-2 target position. **(B1)** Only the first mutation shown in **(A)** has been mutated. **(B2)** The first mutation and the second mutation shown in **(A)** have been mutated. **(B3)** The first mutation, the second mutation, and the third mutation in **(A)** have been mutated. **(B4)** The first mutation, the second mutation, the third mutation, and the fourth mutation shown in **(A)** have been mutated. **(C)** Four mutation sites were selected at the position of crRNA-1 of SFTSV-HB29. **(D)** The SFTSV-HB29 strain plasmid was detected after one, two, three, and four point mutations in the crRNA-2 target position. **(D1)** Only the first mutation shown in **(C)** has been mutated. **(D2)** The first mutation and the second mutation shown in **(C)** have been mutated. **(D3)** The first mutation, the second mutation, and the third mutation in **(C)** have been mutated. **(D4)** The first mutation, the second mutation, the third mutation, and the fourth mutation shown in **(C)** have been mutated.

Through the gene comparison sequence on NCBI, we found that the SFTSV-HB29 strain and the SFTSV-NM1 strain were only four bases apart in the complementary position of crRNA. To verify whether the CRISPR/Cas12a-based SFTSV detection system could be used for the typing of different strains of the same virus, we cloned and obtained SFTSV-HB29 with one, two, three, and four mutation bases in the detection position. The plasmid sequence with four base mutations was the SFTSV-NM1 strain (Figure 4A). The crRNA refers to crRNA-1 (UAAUUUCUACUAAGUGUAGAUAGAAUUGGGGAAUGUUCCCUCCAC; Table 1). Then, we used crRNA-1 for the genotyping of SFTSV strains HB29 and NM1 (Figure 4B). We observed that the fluorescence intensities of the base at the detection position were greatly reduced after mutation; at the same concentration, virus strains with a difference of a few bases were possible to distinguish (Figure 4B). Then, we tried to design four single-base mutations at 2,775 bp of the SFTSV L gene to verify the specificity of the system again (Figure 4C). We used crRNA-2 (UAAUUUCUACUAA GUGUAGAUGAAAAGUUGCUUGUAGCUUUCAUG), primer-F7, and primer-R7 to complete the detection (Table 1). The results showed that the CRISPR/Cas12a SFTSV detection system could achieve single-base typing (Figure 4D).

### Performance of SFTSV CRISPR/Cas12a Assay in Clinical Samples

**Figure 5:**
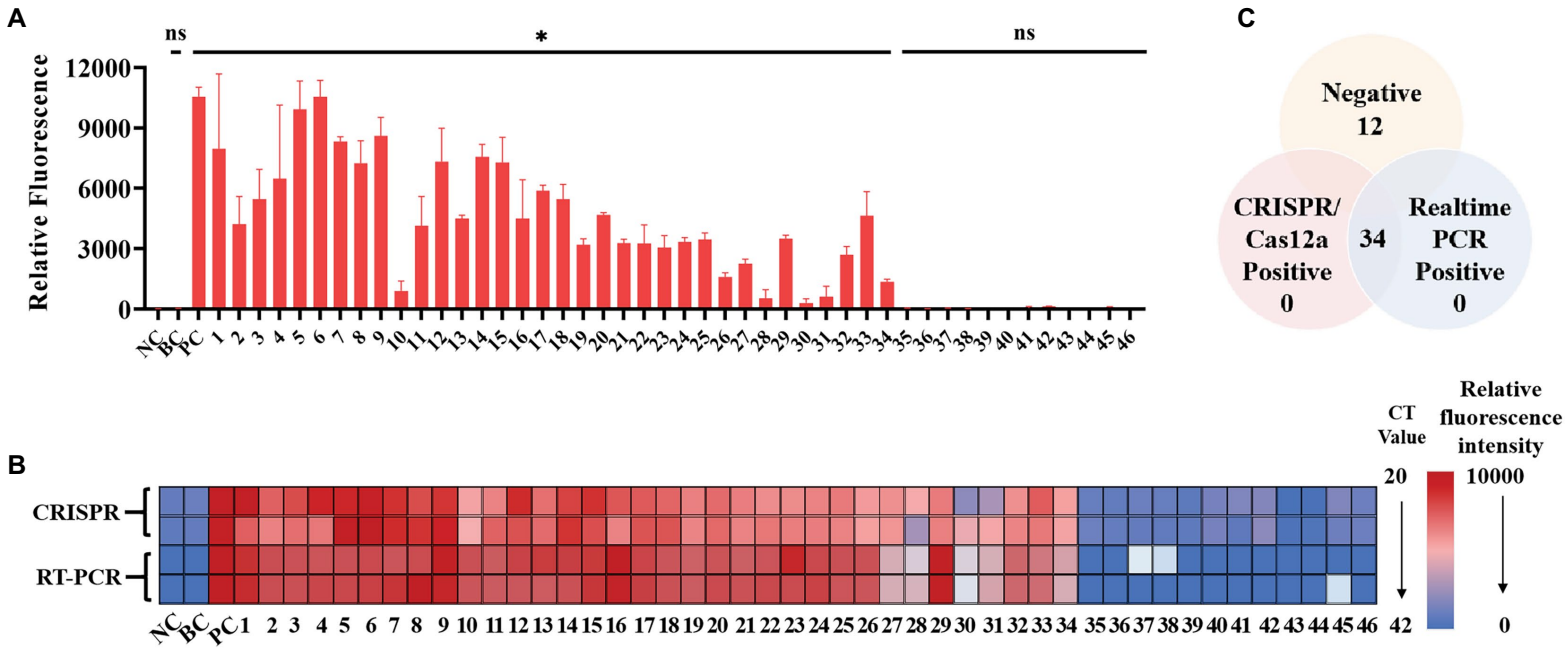
Performance of the SFTSV CRISPR/Cas12a assay on clinical samples. **(A)** The DNA from 46 serum samples was extracted, reverse transcribed, and preamplified with RPA for 20 min. The fluorescence signal was detected by the CRISPR/Cas12a system for 20 min. NC: Negative Control; BC: Blank Control; PC: Positive Control. **(B)** The relative fluorescence value and the Ct value of each clinical sample are displayed by a color scale diagram. NC: Negative Control; BC: Blank Control; PC: Positive Control. **(C)** The Venn diagram shows the consistency between the CRISPR/Cas12a assay and the Q-PCR assay. The unpaired *t* test was applied for the statistical analysis in GraphPad Software Prism 8, *p < 0.05 and ns *p* > 0.05.

A total of 34 patient serum samples and 12 healthy serum samples were used for SFTSV detection with the CRISPR/Cas12a assay. Among these samples, 34 samples were determined using crRNA-1 to be L-gene-positive, and 12 samples were determined using crRNA-1 to be L-negative within 20 min (Figure 5A). As a control for SFTSV detection, Q-PCR was also performed in parallel. The fluorescence value and the Ct value of each sample were analyzed by color scale analysis (Figure 5B) and Wayne diagram analysis (Figure 5C). Compared with Q-PCR, the CRISPR/Cas12a assay correctly identified and differentiated all 34 positive samples and 12 negative samples, showing 100% agreement (Supplementary Figure 2). There was no significant difference between CRISPR/Cas12a and Q-PCR detection of SFTSV.

### Detection of SFTSV With CRISPR/Cas12a Through Immune Osmosis

**Figure 6:**
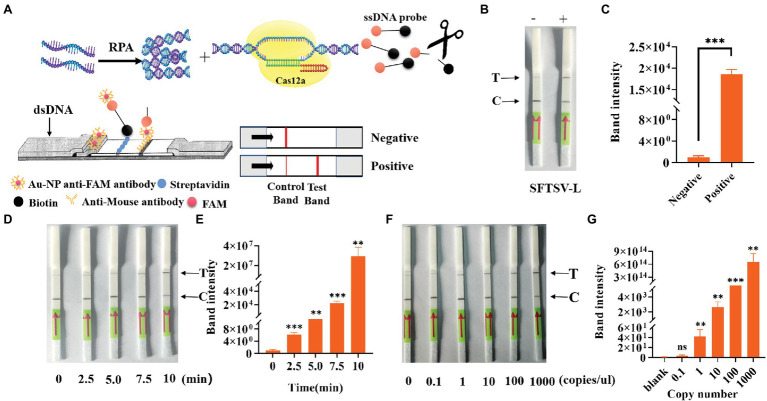
Detection of SFTSV with CRISPR/Cas12a based on immune osmosis. **(A)** Schematic diagram of the SFTSV detection principle of CRISPR/Cas12a combined with immunochromatographic strips. **(B)** The feasibility of CRISPR/Cas12a combined with the immunochromatographic strip system was tested. **(D)** Change trend of the test band over time. **(F)** The sensitivity of CRISPR/Cas12a combined with immunochromatographic strips for the detection of L genes. **(C,E,G)** The visualization of sample test band intensity was quantified by ImageJ and GraphPad. The unpaired *t* test was applied for the statistical analysis in GraphPad Software Prism 8, *p < 0.05; **p < 0.01; ***p < 0.001; and ns *p* > 0.05.

To simplify the field test equipment, we combined the CRISPR/Cas12a system with the immunochromatographic strips. We chose crRNA-1 for immunochromatographic strip detection. As shown in Figure 6A, gold nanoparticle aggregation, immune osmosis principles, and the FAM and biotin-labeled ssDNA probe were used to verify the collateral nuclease activity of CRISPR complexes. After adding the CRISPR/Cas12a system, the FAM-labeled probe was combined with the anti-FAM antibody, and the biotin bound to streptavidin. This resulted in the aggregation of Au-NP, which represented the control band. When the probe was cut, the free probe continued to penetrate upward until the anti-FAM antibody bound to the secondary antibody, which also resulted in Au-NP aggregation, representing a test band and a positive result. By testing the system, we found that the immunochromatographic strips helped to stably detect 1,000 copies/μl sample of the L gene (Figure 6B) in 10 min. We obtained the same conclusion by analyzing test band density with ImageJ and drawing a histogram, finding that the band density of the positive sample was significantly different from that of blank group (Figure 6C). Furthermore, we tested the change of the depth of the strip over time (Figure 6D); the results showed that a longer duration was associated with a deeper test band of the strip. We also used standards to explore the test strip limits and found a clear distinction for the 1 copy/μl SFTSV standard (Figure 6F). Similarly, the test band density was analyzed by ImageJ, and the histogram graph analysis showed that the immunochromatographic strip could stably detect the clinical samples, which was in agreement with Figure 3 (Figures 6E,G). In general, these results show that the CRISPR/Cas12a assay can be stably applied to the immunochromatographic strip detection system, thereby reducing the technical requirements for instruments and operators.

## Discussion

In this study, we developed an efficient and convenient SFTSV detection method combining RPA and the CRISPR/Cas12a system for the early diagnosis of SFTSV infection in patients. We detected the L gene from the SFTSV genome using the CRISPR/Cas12a method, and the CRISPR/Cas12a method was combined with immunochromatographic strips to simplify the requirements for field testing instruments.

Fever with thrombocytopenia syndrome caused by SFTSV infection poses a great threat to public health. Unfortunately, no effective therapeutic drugs or vaccines currently prevent SFTSV infection. Therefore, early detection of SFTSV is necessary. At present, the detection methods for SFTSV mainly focus on virus isolation, real-time PCR, and serological detection. Moreover, although virus isolation and identification is a great way to determine the virus, the trial period is time-consuming, up to 1–2 weeks. Real-time PCR is a very sensitive detection method, but it requires expensive equipment and professional laboratory techniques. The sensitivity and specificity of serological detection often do not meet our requirements. Therefore, none of the above three commonly used detection methods can be used for effective and rapid on-site detection.

Compared with traditional amplification detection methods, CRISPR/Cas12a detection of the SFTSV L gene requires relatively simple equipment conditions, which greatly reduce the cost of detection, shorten the detection duration, and facilitate on-site pathogen detection. We used a portable fluorescence isothermal amplification instrument, which has advantages of its small size, easy operation, and automatic positive signal detection. Unlike other detection methods, CRISPR/Cas12a can effectively achieve the typing and detection of SFTSV (Figure 4). Combined with test strips, this method can even be performed directly in the field, without machine readings, and the results can be judged by the naked eye (Figure 6). Its sensitivity is equal to or even better than of Q-PCR. Regardless of how it is viewed, this is a potentially effective detection method for SFTSV.

The only minor issues associated with the SFTSV detection method based on CRISPR/Cas12a were as follows. The operator needed to open the cover three times during the reaction, which increased the possibility of false-positive results. This was indeed a huge problem faced by CRISPR-based detection systems, but the activity of Cas12a was still affected by temperature and ion concentration, thereby possibly affecting the detection sensitivity. Moreover, one-step detection may affect enzyme activity. Therefore, to optimize Cas12a and amplification enzyme activity, reducing its working temperature requirements or designing and modifying reaction tubes and detection instruments may effectively solve this problem, to achieve open-tube operation in the future. In summary, we developed an SFTSV detection method based on RPA and the CRISPR/Cas12a system that can be used for rapid and ultrasensitive SFTSV diagnosis, providing a powerful and effective tool for on-site detection.

## Data Availability Statement

The raw data supporting the conclusions of this article will be made available by the authors, without undue reservation.

## Ethics Statement

The studies involving human participants were reviewed and approved by Ethics Review Committee of Tianjin University, Tianjin University. The ethics committee waived the requirement of written informed consent for participation.

## Author Contributions

MH: data curation, formal analysis, investigation, methodology, visualization, and writing – original draft. SL: data curation, formal analysis, investigation, and visualization. YX: data curation and methodology. AL: funding acquisition and writing – review and editing. WW: project administration. ML: methodology. GN: data curation. ZW: conceptualization, project administration, resources, and supervision. TW: conceptualization, funding acquisition, project administration, resources, supervision, and writing – review and editing. All authors contributed to the article and approved the submitted version.

## Funding

This work was supported by the National Key Research and Development Program of China (2017YFA0205102) and the National Science Foundation of China (32170144).

## Conflict of Interest

The authors declare that the research was conducted in the absence of any commercial or financial relationships that could be construed as a potential conflict of interest.

## Publisher’s Note

All claims expressed in this article are solely those of the authors and do not necessarily represent those of their affiliated organizations, or those of the publisher, the editors and the reviewers. Any product that may be evaluated in this article, or claim that may be made by its manufacturer, is not guaranteed or endorsed by the publisher.
