# Supplementary material for: CRISPR/Cas12a Technology Combined With RPA for Rapid and Portable SFTSV Detection
Source: Front Microbiol. 2022 Jan 25;13:754995. doi: 10.3389/fmicb.2022.754995 (PMC8822122; doi:10.3389/fmicb.2022.754995)
Supplement: Supplementary file 1 [file Table_1.doc]

Supplementary Material

## Supplementary Figures


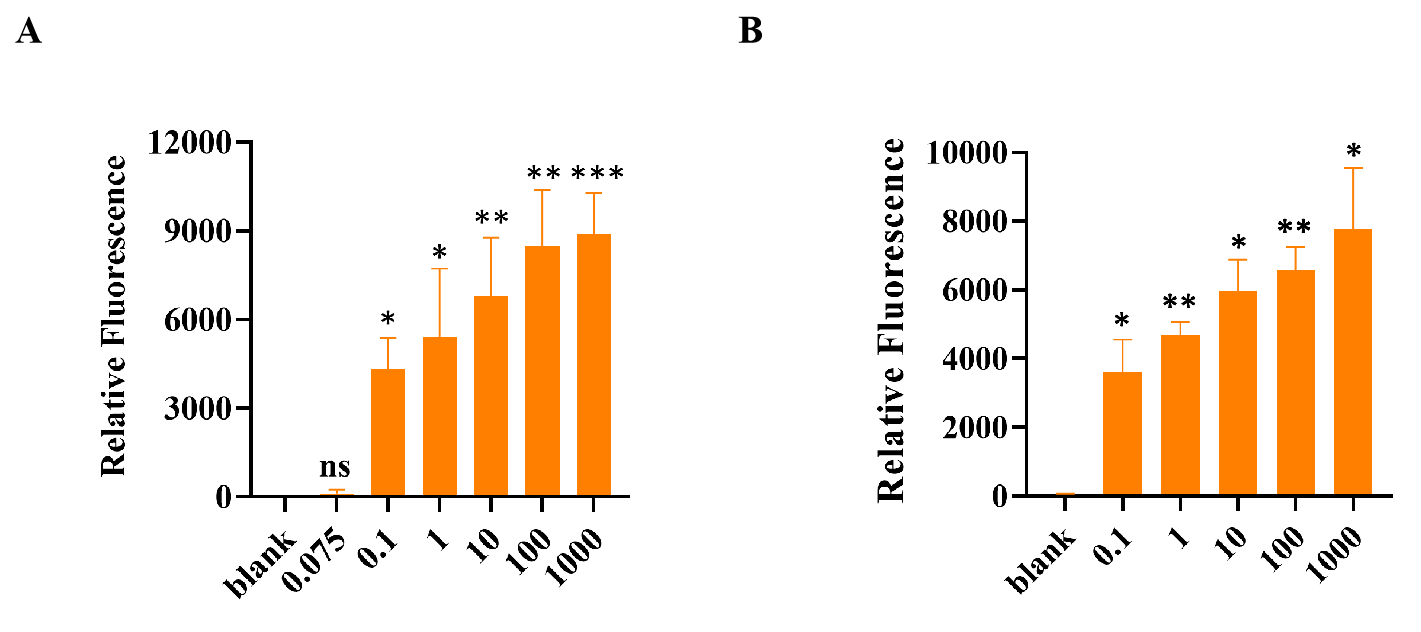


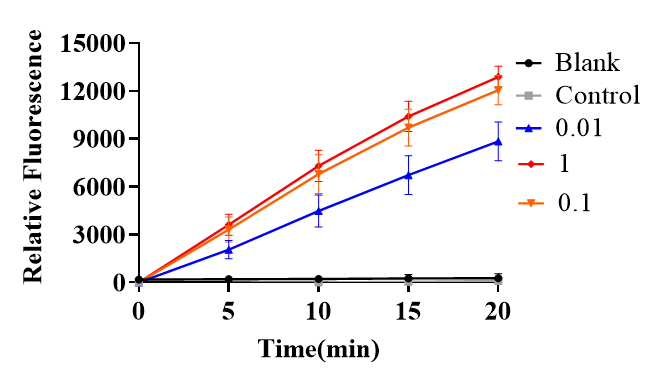
**Supplementary Figure 1**. Significance analysis results. A. The results of the significance analysis of Figure 3C. B. The results of the significance analysis of Figure 3D. The unpaired t test was applied for the statistical analysis in GraphPad Software Prism 8, *p<0.05;**p<0.01; ***p<0.001; and ns p>0.05.

**Supplementary Figure 2.** The CRISPR/Cas12a system detects infected cell samples. We infected Vero cells with SFTSV at an MOI of 0.01, 0.1, and 1. After 24 h, the cells were harvested and RNA was extracted and tested.


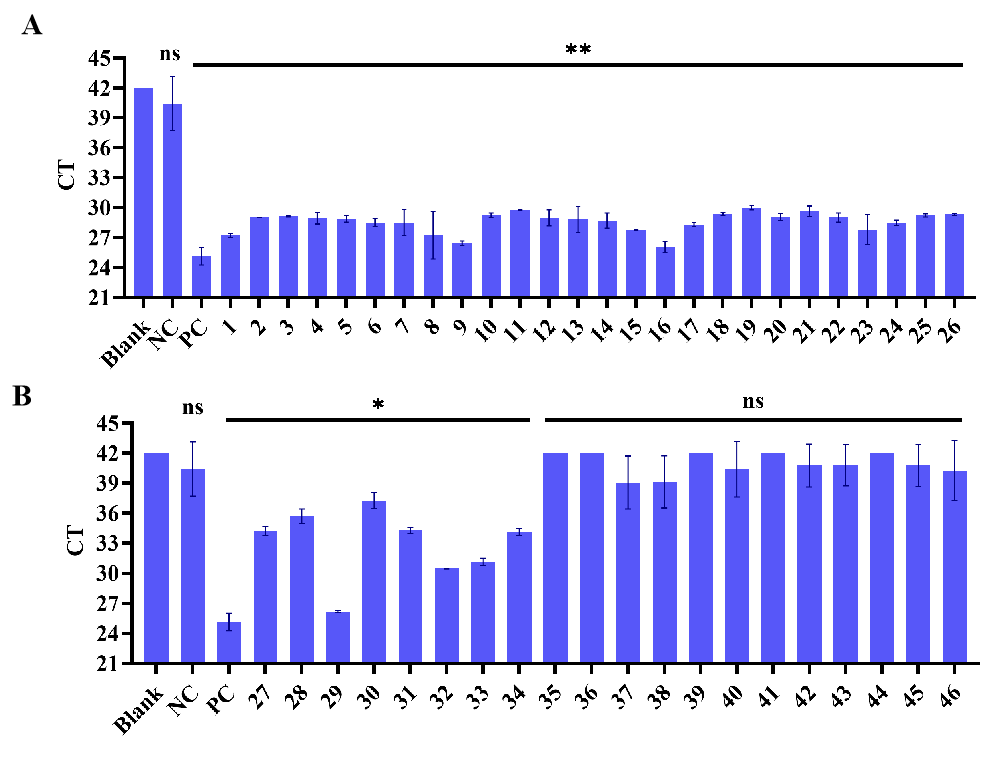


**Supplementary Figure 3.** Q-PCR tests results of clinical samples. The unpaired t test was applied for the statistical analysis in GraphPad Software Prism 8, *p<0.05;**p<0.01; and ns p>0.05.
